# Synergistic effects of *Cinnamomum cassia* L. essential oil in combination with polymyxin B against carbapenemase-producing *Klebsiella pneumoniae* and *Serratia marcescens*

**DOI:** 10.1371/journal.pone.0236505

**Published:** 2020-07-23

**Authors:** Nathalie Gaebler Vasconcelos, Júlio Henrique Ferreira de Sá Queiroz, Késia Esther da Silva, Paulo César de Paula Vasconcelos, Julio Croda, Simone Simionatto

**Affiliations:** 1 Laboratório de Pesquisa em Ciências da Saúde, Universidade Federal da Grande Dourados—UFGD, Dourados, Mato Grasso do Sul, Brazil; 2 Hospital Universitário de Dourados, Universidade Federal da Grande Dourados—UFGD, Dourados, Mato Grosso do Sul, Brazil; 3 Fundação Oswaldo Cruz, Campo Grande, Mato Grosso do Sul, Brazil; 4 Universidade Federal do Mato Grosso do Sul, Campo Grande, Mato Grosso do Sul, Brazil; Northern Arizona University, UNITED STATES

## Abstract

Multidrug resistance prompts the search for new sources of antibiotics with new targets at bacteria cell. To investigate the antibacterial activity of *Cinnamomum cassia* L. essential oil (CCeo) alone and in combination with antibiotics against carbapenemase-producing *Klebsiella pneumoniae* and *Serratia marcescens*. The antimicrobial susceptibility of the strains was determined by Vitek^®^ 2 and confirmed by MALDI-TOF/TOF. The antibacterial activity of CCeo and its synergism with antibiotics was determined using agar disk diffusion, broth microdilution, time-kill, and checkboard methods. The integrity of the bacterial cell membrane in *S*. *marcescens* was monitored by protein leakage assay. CCeo exhibited inhibitory effects with MIC = 281.25 μg.mL^-1^. The association between CCeo and polymyxin B showed a decrease in terms of viable cell counts on survival curves over time after a 4 hour-treatment with a FIC index value of 0.006. Protein leakage was observed with increasing concentrations for CCeo and CCeo + polymyxin B treatments. CCeo showed antibacterial activity against the studied strains. When associated with polymyxin B, a synergistic effect was able to inhibit bacterial growth rapidly and consistently, making it a potential candidate for the development of an alternative treatment and drug delivery system for carbapenemase-producing strains.

## Introduction

*Klebsiella pneumoniae* and *Serratia marcescens* are able to acquire and share their resistance genes, leading to multidrug resistance, which can result in severe infections. One of the most important therapeutic challenges posed by Enterobacterales is their resistance to carbapenems [1]. Carbapenemase encoding-genes are located on self-conjugative plasmids, capable of disseminating among bacteria, resulting in the spread of resistance [2]. Clinical options for hard-to-treat infections include tigecycline, polymyxins, aminoglycosides, and fosfomycin [1]. However, the use of these antibiotics is limited because of their toxicity and pharmacokinetic properties [3].

*Serratia* is a genus naturally resistant to polymyxins. It has a modified lipopolysaccharide (LPS) where the normally encountered Enterobacterales cationic phosphate groups are substituted for 4-amino-4-deoxy-L-arabinose (L-Ara4N) or phosphoethanolamine [4]. The cationic substitution of the phosphate groups by L-Ara4N [5] is the most common modification. This increases the net negative charge of lipid A of LPS results in polymyxins being repelled by bacterial cells [4].

The operon *arnBCADTEF-pmrE* (also called *pmrHFIJKLM-ugd*) mediates the synthesis and transfer of L-Ara4N to lipid A [4–6]. The inactivation of the *arnB* and *arnC* genes can result in polymyxin sensitivity in *S*. *marcescens* [4]. The *arnBCADTEF-pmrE* operon appears to be constitutively expressed in intrinsically resistant bacteria [6]. Due to widespread distribution of carbapenemase-producing bacteria across the world and the limitations of current treatments for its infections, new therapeutic alternatives are needed [7]. Thus, novel sources of chemicals with antimicrobial activity against multidrug resistance strains are required.

Essential oils are potential sources of antimicrobial compounds. Several studies have demonstrated the antibacterial, antifungal, antiviral, and antiparasitic properties of essential oils from plants [8, 9]. These essential oils and their components have a variety of cellular targets, particularly the membrane and cytoplasm, and may alter the morphology of cells [9], making them an interesting area of research. Essential oils are promising novel functional excipients in the development of new drug delivery systems [10]. Excipients are considered as essential constituents able to guarantee the performance of a drug and optimize the attainment of its therapeutic effects [11]. Essential oils will need to be studied in order to make the best use of their antibacterial activities and to reduce the concentrations of antibiotics required to achieve an antibacterial effect. The antimicrobial activities of *Cinnamomum cassia* and its essential oil have been reported in bacteria sensitive to antibiotics [12, 13]. The main compound responsible for antimicrobial activity in cinnamon bark essential oil is cinnamaldehyde, which is also the dominant substance [7].

This study describes the antibacterial efficacy of *C*. *cassia* L. essential oil (CCeo) against carbapenemase-producing *K*. *pneumoniae* and *S*. *marcescens* and its synergistic effects when used in combination with antibiotics.

## Materials and methods

### Essential oil

The essential oil used in this study was *C*. *cassia*, a yellow to yellow brown clear oily liquid, extracted from the leaves, bark, and branches by steam distillation, with a density of 1.053 g/cm^3^, originating from China and acquired commercially from Ferquima (Vargem Grande Paulista, SP, Brazil) under the product name of “Essential Oil of Cinnamon Cassia” (CAS number: 84961-46-6; batch number: 217). Trans-cinnamaldehyde 99% (CAS number: 14371-10-9) was obtained from Sigma-Aldrich (St. Louis, MO, USA). According to the chromatographic technical report provided by the supplier, cinnamaldehyde was the most prevalent compound (87.6%), followed by α-humulene (3.1%), γ-elemene (2.5%), borneol (1.5%), cinnamic acid (0.7%), benzaldehyde (0.5%), eugenol (0.4%), and other minor components (3.7%) (S1 Fig).

### Bacterial strains

Carbapenemase*-*producing *K*. *pneumoniae* (KP-KPC) and *S*. *marcescens* (SM-KPC) were collected and isolated from human specimens by rectal swab and urine sample, respectively. Samples were taken from patients over 50 years old, hospitalized in different wards at a tertiary hospital in Brazil’s Midwestern region and are representative of the carbapenemase-producing Gram-negative bacteria group. Bacterial strains evaluated in this study were representative from predominant clonal type (33 KPC-producing *K*. *pneumoniae* and 6 KPC-producing *S*. *marcescens*), previously characterized [14, 15].

Selected isolates were maintained at –70°C, then sub-cultured on MacConkey agar for 24 h before testing. These isolates were identified and characterized according to their sensibility to antibiotics by Vitek^®^ 2 (bioMérieux, Hazelwood, MO, USA). Their minimal inhibitory concentrations (MIC) were confirmed by broth microdilution. Preliminary screening for the presence of carbapenemase was performed using the modified Hodge test [16] and confirmed by matrix-assisted laser desorption ionization-time of flight mass spectrometry (MALDI-TOF/TOF) using a Microflex LT spectrometer (Bruker Daltonics, Billerica, MA, USA) [17].

### Detection of resistance genes

Polymerase chain reaction (PCR) was performed to confirm the presence of the genes *bla*_CTX-M-1-like_, *bla*_CTX-M-2-like_, *bla*_CTX-M-8-like_, *bla*_CTX-M-14-like_, *bla*_GES-like_, *bla*_GIM-like_, *bla*_IMP-10_, *bla*_IMP-like_, *bla*_KPC-2_, *bla*_NDM-like_, *bla*_OXA-23_, *bla*_OXA-48-like_, *bla*_SHV-like_, *bla*_SIM-like_, *bla*_SME-like_, *bla*_SPM-like_, *bla*_TEM-like_, and *bla*_VIM-like_ in KP-KPC and SM-KPC strains. Total DNA of KP-KPC and SM-KPC isolates was extracted using the QIAamp DNA minikit^®^ (Qiagen, Courtaboeuf, France) and used as the template in PCR experiments. Amplification was performed using specific primers and cycling parameters, according to the target sequence. Genomic DNA was quantified using a NanoDrop TM 1000 spectrophotometer (Thermo Fischer Scientific, Waltham, Massachusetts, MA, USA). Polymerase chain reactions (PCRs) were prepared as standard 25 μL volumes comprising 12.5 μL of 2 x Master mix [0.05 U/μL Taq DNA polymerase, reaction buffer, 4 mM MgCl_2_, 0.4 mM of each dATP, dCTP, dGTP and dTTp], 0.25 μL of each primer (1 μL), 1 μL template DNA (20−30 ng/μL) and 11 μL nuclease free water. PCR was performed using a Thermal cycler (model C1000 Touch) supplied by BIO-RAD, California, USA. Sequences of the primers used to the amplification of *bla* genes are shown in Table 1. The PCR products were analyzed in horizontal electrophoresis using a 1% agarose gel and a 50-bp DNA ladder (Ludwig, Brazil) as a molecular marker, then PCR products were visualized by UV light at 336 nm.

**Table 1:** Sequences of the primers used to the amplification of *bla* genes.

Primer	Sequence (5'-3')	Reference	Product length
*bla*_CTX-M-1-like_	F: CGCTTTGCGATGTGCAG	[18]	512 bp
	R: ACCGCGATATCGTTGGT		
*bla*_CTX-M-2-like_	F: CGACGCTACCCCTGCTATT	[19]	552 bp
	R: CCAGCGTCAGATTTTTCAGG		
*bla*_CTX-M-8-like_	F: TCGCGTTAAGCGGATGATGC	[19]	666 bp
	R: AACCCACGATGTGGGTAGC		
*bla*_CTX-M-14-like_	F: TACAGCCCTTCGGCGATGA	[18]	874 bp
	R: GGTGACAAAGAGAGTGCAACGGAT		
*bla*_GES-like_	F: GTTTTGCAATGTGCTCAACG	[20]	371 bp
	R: TGCCATAGCAATAGGCGTAG		
*bla*_GIM-like_	F: TCGACACACCTTGGTCTGAA	[20]	477 bp
	R: AACTTCCAACTTTGCCATGC		
*bla*_IMP-10_	F: CCAAACYACTASGTTATC	[18]	188 bp
	R: GAATAGRRTGGCTTAAYTCTC		
*bla*_IMP-like_	F: GGAATAGAGTGGCTTAA(C/T)TCTC	[20]	232 bp
	R: GGTTTAA(C/T)AAAACAACCACC		
*bla*_KPC-2_	F: TCTGGACCGCTGGGAGCTGG	[21]	399 bp
	R: TGCCCGTTGACGCCCAATCC		
*bla*_NDM-like_	F: GGTTTGGCGATCTGGTTTTC	[20]	621 bp
	R: CGGAATGGCTCATCACGATC		
*bla*_OXA-23_	F: GATCGGATTGGAGAACCAGA	[22]	501 bp
	R: ATTTCTGACCGCATTTCCAT		
*bla*_OXA-48-like_	F: TTGGTGGCATCGATTATCGG	[18]	798 bp
	R: GAGCACTTCTTTTGTGATGGC		
*bla*_SHV-like_	F: CTTGACCGCTGGGAAACGG	[18]	200 bp
	R: AGCACGGAGCGGATCAACGG		
*bla*_SIM-like_	F: TACAAGGGATTCGGCATCG	[20]	570 bp
	R: TAATGGCCTGTTCCCATGTG		
*bla*_SME-like_	F: TATGGAACGATTTCTTGGCG	[23]	300 bp
	R: CTCCCAGTTTTGTCACCTAC		
*bla*_SPM-like_	F: AAAATCTGGGTACGCAAACG	[20]	271 bp
	R: ACATTATCCGCTGGAACAGG		
*bla*_TEM-like_	F: CCCTTATTCCCTTTYTTGCGG	[18]	680 bp
	R: AACCAGCCAGCCWGAAGG		
*bla*_VIM-like_	F: GATGGTGTTTGGTCGCATA	[20]	390 bp
	R: CGAATGCGCAGCACCAG		

bp: base pairs; F: forward primer; R: reverse primer.

### Antibacterial assays of CCeo

The antibacterial activity of raw CCeo was determined using the standard method of agar disk diffusion. Polymyxin B and tigecycline were used as the positive standards. Filter paper disks of 6 mm diameter, containing 10 μl of the CCeo were evaluated against KP-KPC and SM-KPC. After 30 min at room temperature, the dishes were incubated at 37 ± 1°C, for 24 h and the diameter of the inhibition zone formed was measured in millimeters [24]. The test was performed in duplicate and the means of the values obtained were used to classify the bacterium as sensitive (≥ 10 mm) or resistant (< 10 mm) [25]. The MIC of CCeo and trans-cinnamaldehyde (TC) were determined by broth microdilution, as described by Cavalcanti et al. [26] and was determined by resazurin colorimetric assay. CCeo was diluted with distilled water and 0.5% Tween-80.

### Synergy tests

The agar disk diffusion assay was performed using the Kirby–Bauer methods for synergy screening. Here, CCeo was evaluated alone and in combination several antibiotics (amikacin, ciprofloxacin, imipenem (IPM), piperacillin/tazobactam, polymyxin B (POL), and tigecycline (Sigma-Aldrich). Antibiotic disks were placed on a Müller-Hinton plate inoculated with KP-KPC or SM-KPC and 10 μl of the CCeo were added in the antibiotic disk [24]. After incubation, the inhibition zone of the combination was comparatively examined with the independent test samples. Synergistic interactions were evaluated if the inhibition zone in the combination was larger than 2 mm or more in comparison to either antibiotics or CCeo alone [27]. The test was performed in duplicate.

### Time-kill

The time-kill method was performed using the broth macrodilution technique [28]. CCeo alone and in combination with antibiotic (imipenem or polymyxin B) was tested against the KP-KPC and SM-KPC strains. The concentration of each antimicrobial agent tested represented the MIC value. The polymyxin B concentration used as MIC for SM-KPC was 1 μg.mL^-1^, which was the same as the polymyxin B MIC for KP-KPC. The time-kill studies were performed with approximately 1.5 × 10^6^ colony forming units (CFU).mL^-1^ in a final volume of 3.2 mL, verified with a spectrophotometer by Vitek^®^ 2 (bioMérieux). The tubes were shaken periodically and incubated at 37°C. Samples were obtained after 0, 4, 8, 12, 16, 20, and 24 h of incubation. At each sample time, 1 μl was withdrawn from each tube using a sterile loop and seeded in MacConkey agar plates. The plates were incubated for 24 h at 37°C and the colony counts were determined. The resulting colony count of the samples treated with the combination was compared to the samples treated with the most effective single agent. A decrease in colony count of 100-fold or more was considered synergism, while an increase in colony count of 100-fold or more was considered antagonism. Changes that were limited to 10-fold were defined as additive or indifferent [29]. A negative control of BHI was used for the bacterial strain, and saline was used as a sterility control. Polymyxin B and gentamicin (GEN) were used as the positive controls for KP-KPC and SM-KPC strains, respectively. The test was performed in triplicate.

### Checkboard

To evaluate the potentiating effect of CCeo, combinations of CCeo and polymyxin B were assessed against the KP-KPC and SM-KPC strains. The concentration of each antimicrobial agent ranged from 1/32 to 2 × MIC. The polymyxin B concentration used as MIC for SM-KPC was 1 μg.mL^-1^. Using a microtiter plate, two-fold serial dilutions of the oils were prepared in the horizontal rows and two-fold serial dilutions of the antibiotics were prepared in the vertical rows. The plates were prepared well-by-well to obtain a single plate in which both antimicrobial agents were cross-diluted. The inoculum used was 1.5 × 10^6^ CFU.mL^-1^. Resazurin was used to indicate viable bacteria. The fractional inhibitory concentration (FIC) of each combination was then calculated as the ratio of MIC of the antimicrobial agent in combination versus the MIC of the antimicrobial agent alone [29]. The ΣFIC is then calculated for each test sample independently.

The FIC index was calculated as follows: ΣFIC = FIC^(*i)^ + FIC^(*ii)^. The results were interpreted as either synergistic (ΣFIC ≤ 0.5), additive (0.5 < ΣFIC < 1.0), noninteractive (1.0 < ΣFIC ≤ 4.0), or antagonistic effects (ΣFIC > 4.0) [29]. All experiments were carried out in duplicate as two independent experiments and the calculated FIC indexes were averaged. A negative control of BHI was used for the bacterial strains and saline was used as a sterility control. The sterility of CCeo, polymyxin B, and water was assessed. A second negative control, in the form of 0.5% Tween-80, was also used.

### Determination of cell membrane integrity

The integrity of the bacterial cell membrane was monitored by the release of proteins from the cell into the supernatant. Carbapenemase-producing SM-KPC was incubated in MacConkey agar at 37°C for 24 h. Three groups with bacterial inoculum in the logarithmic growth phase (1.5 × 10^6^ CFU.mL^-1^) were placed in a 96-well microplate, treated with the combination CCeo + polymyxin B, with 0.5, 1, and 2 × the MIC of each agent. The microplate was incubated at 37°C for 0, 1, 2, and 4 h. Then, 1 μL aliquot of CCeo + polymyxin B treatment (concentration of MIC) were seeded in Müller-Hinton agar to confirm the viability of the strain at each time-point. The content of each microplate well was then centrifuged at 2,500 rpm for 5 min at 4°C. The concentration of the proteins released from the cytoplasm was then assessed in the supernatant using the Pierce^TM^ BCA Protein Assay kit. The optical absorbance (OD) was read at 490 nm using the iMark^TM^ Microplate Absorbance Reader (Bio-Rad, São Paulo, SP, Brazil). The experiment was performed in duplicate. Saline with bacterium was used as negative control. CCeo and polymyxin B were diluted with distilled water.

### Statistical analysis

The experimental data derived from time–kill and determination of cell membrane integrity assays were analyzed using linear regression slopes comparison—GraphPad Prism 6.01 (GraphPad Inc., San Diego, CA, USA). For time kill, log CFU values were plotted against time for each antibiotic. The kill rate was determined at different time intervals (0, 4, 8, 12, 16, 20 and 24 h), undertaking a linear regression to find the slope for each antimicrobial to test the difference between the slopes and intercepts. For determination of cell membrane integrity assay, concentration of protein was plotted against antimicrobial agent’s concentration. P value < 0.05 was considered significant.

### Molecular analysis of *16S* and *arnB* genes in SM-KPC strain

Reverse transcriptase quantitative polymerase chain reaction (RT-qPCR) analysis was performed to measure the changes in the expression levels of the small subunit 16S of ribosomal RNA during treatment with CCeo in association with polymyxin B. Total RNA was isolated from SM-KPC before and after treatment (at MIC values), transformed into cDNA using the iScript^TM^ cDNA Synthesis kit (Bio-Rad). The cDNA was then subjected to RT-qPCR using the primers pairs 16S forward (16SF) and 16S reverse (16SR) (Table 2) and SYBR^®^ Green JumpStart^TM^ Taq ReadyMix^TM^ (Sigma-Aldrich). The reactions were performed using the following reagents: SYBR Green mix, 16SF primer, and 16SR primer (10 μM), and template (20 ng), using double-distilled water to obtain the reaction final volume. RT-qPCR cycling was carried out using the CFX96 Touch^TM^ Real-Time PCR Detection System (Bio-Rad) as follows: initial denaturation at 94°C for 2 min, then 40 cycles of 94°C (15 sec) and 60°C (1 min). All PCR assay reactions were performed in triplicate. Negative controls were included with each PCR assay. Following amplification, the results were analysed on the PCR computer using the CFX Manager ver. 3.1.15 software. Once amplification was completed, the level of amplification was reported by the software as the mean cycle threshold (CT) value of the replicate samples. A sample was considered positive by real-time RT-qPCR and expressed the 16S gene if the CT value was above the threshold value before the 40 cycles of the PCR reaction and below the negative control CT.

**Table 2:** Primers used for amplification of *arnB* and *16S* genes.

Primer	Sequence 5´-3´	Nucleotide position	Product length
***arnB*F**	CCAAAGCGATTGTTCCGGTG	365–384	169 bp
***arnB*R**	AAAGAGAAAATCGCCGTGCC	609–590	
***16S*F**	ATTCCAGGTGTAGCGGTGAA	646–665	238 bp
***16S*R**	TGAGTTTTAACCTTGCGGCC	883–864	

*16S*F: *16S* gene forward primer; *16S*R: *16S* gene reverse primer; *arnB*F: *arnB* gene forward primer; *arnB*R: *arnB* gene reverse primer; bp: base pairs.

Reverse transcriptase polymerase chain reaction (RT-PCR) was also performed on the *arnB* genes in the SM-KPC strain before and after treatment with CCeo + polymyxin B or polymyxin B alone [14, 15]. The primers *arnB*F and *arnB*R were designed based on the gene sequences of *S*. *marcescens* subsp. *marcescens* Db11 and *S*. *marcescens* 2880STDY5682918 (GenBank Accession No. HG326223.1, NZ_FCJC01000002.1) (Table 2). Total RNA was isolated and transformed into cDNA using iScriptTM cDNA Synthesis kit (Bio-Rad) and assessed by RT-PCR (Lin2014). The PCR products were examined by 1% agarose gel electrophoresis and ethidium bromide staining.

### Ethical approval

This study was conducted with the approval of the Research Ethics Committee from the Universidade Federal da Grande Dourados (Process No. 877.292/2014).

## Results

The isolates showed resistance to carbapenems. Sensitivity was observed only to polymyxin B for KP-KPC and amikacin and gentamicin for SM-KPC (Table 3). The strains were identified as carbapenemase-producers by modified Hodge test and MALDI-TOF/TOF. PCR amplification showed that the *bla*_KPC-2_ gene was present in KP-KPC, and *bla*_KPC-2_, *bla*_IMP-10_, and *arnB* in SM-KPC. The presence of *bla*_CTX-M-1-like_, *bla*_CTX-M-2-like_, *bla*_CTX-M-8-like_, *bla*_CTX-M-14-like_, *bla*_GES-like_, *bla*_GIM-like_, *bla*_IMP-like_, *bla*_NDM-like_, *bla*_OXA-23_, *bla*_OXA-24,_ *bla*_OXA-48,_ *bla*_OXA-48-like_, *bla*_OXA-51,_ *bla*_OXA-58,_ *bla*_SHV-like_, *bla*_SIM-like_, *bla*_SME-like_, *bla*_SPM-like_, *bla*_TEM-like_, and *bla*_VIM-like_ genes was not detected.

**Table 3:** Sensitivity of bacterial strains to the action of different antibiotics (results expressed in μg.mL^-1^) assessed by Vitek^®^ 2.

Antibiotics	MIC (Interpretation)	
	KP-KPC	SM-KPC
Amikacin	> 32 (R)	16 (S)
Ampicillin	> 16 (R)	> 16 (R)
Aztreonam	> 16 (R)	> 16 (R)
Cefoxitin	> 16 (R)	> 16 (R)
Ceftazidime	16 (R)	8 (R)
Cefepime	> 16 (R)	8 (R)
Ciprofloxacin	> 2(R)	> 2 (R)
Ertapenem	> 4 (R)	> 4 (R)
Gentamicin	> 8 (R)	≤ 2 (S)
Imipenem	> 8 (R)	> 8 (R)
Levofloxacin	> 4 (R)	> 4 (R)
Meropenem	> 8 (R)	> 8 (R)
Nitrofurantoin	> 64 (R)	> 64 (R)
Piperacillin/Tazobactam	> 64/4 (R)	64/4 (R)
Polymyxin B	≤ 1 (S)	(IR)
Tigecycline	2 (I)	4 (R)
Sulfamethoxazole/Trimethoprim	> 4/76 (R)	≤ 1/19 (S)

KP-KPC: carbapenemase-producing *K*. *pneumoniae*; MIC: minimal inhibitory concentration; SM-KPC: carbapenemase-producing *S*. *marcescens*; (S): susceptibility; (I): intermediate; (R): resistance; (IR): intrinsic resistance.

CCeo exhibited an inhibitory effect against KP-KPC and SM-KPC strains, with MICs of 281.25 μg.mL^-1^. The MIC of trans-cinnamaldehyde was evaluated and found to be the same as the MIC of CCeo for both strains. The initial synergism trial using the disk diffusion method showed the potentiation of imipenem and polymyxin B when associated with CCeo. The PCR results, the disk diffusion, and the MIC of CCeo against KP-KPC and SM-KPC strains and the synergism effect are shown in Table 4.

**Table 4:** Antibacterial action of CCeo and trans-cinnamaldehyde using disk-diffusion and broth microdilution and synergism between CCeo and antibiotics using disk-diffusion as screening.

Method	Antimicrobial agent	Bacteria	
		KP-KPC	SM-KPC
**MIC**	CCeo	25 mm (S)	16 mm (S)
	TC	281.25 μg.mL^-1^	281.25 μg.mL^-1^
**Disk-diffusion**	CCeo	281.25 μg.mL^-1^	281.25 μg.mL^-1^
	IPM	0 mm	0 mm
	CCeo + IPM	25 mm *	20 mm *
	POL	20 mm	14 mm
	CCeo + POL	2 mm *	6 mm *

CCeo: *Cinnamomum cassia* essential oil; IPM: imipenem; IR: intrinsic resistance; KP-KPC: carbapenemase-producing *K*. *pneumoniae*; MIC: minimum inhibitory concentration; POL: polymyxin B; S: susceptibility; SM-KPC: carbapenemase-producing *S*. *marcescens*; TC: trans-cinnamaldehyde

*: diameter of inhibition zone in mm of CCeo + antibiotic minus diameter of inhibition zone of antibiotic.

**Fig 1:**
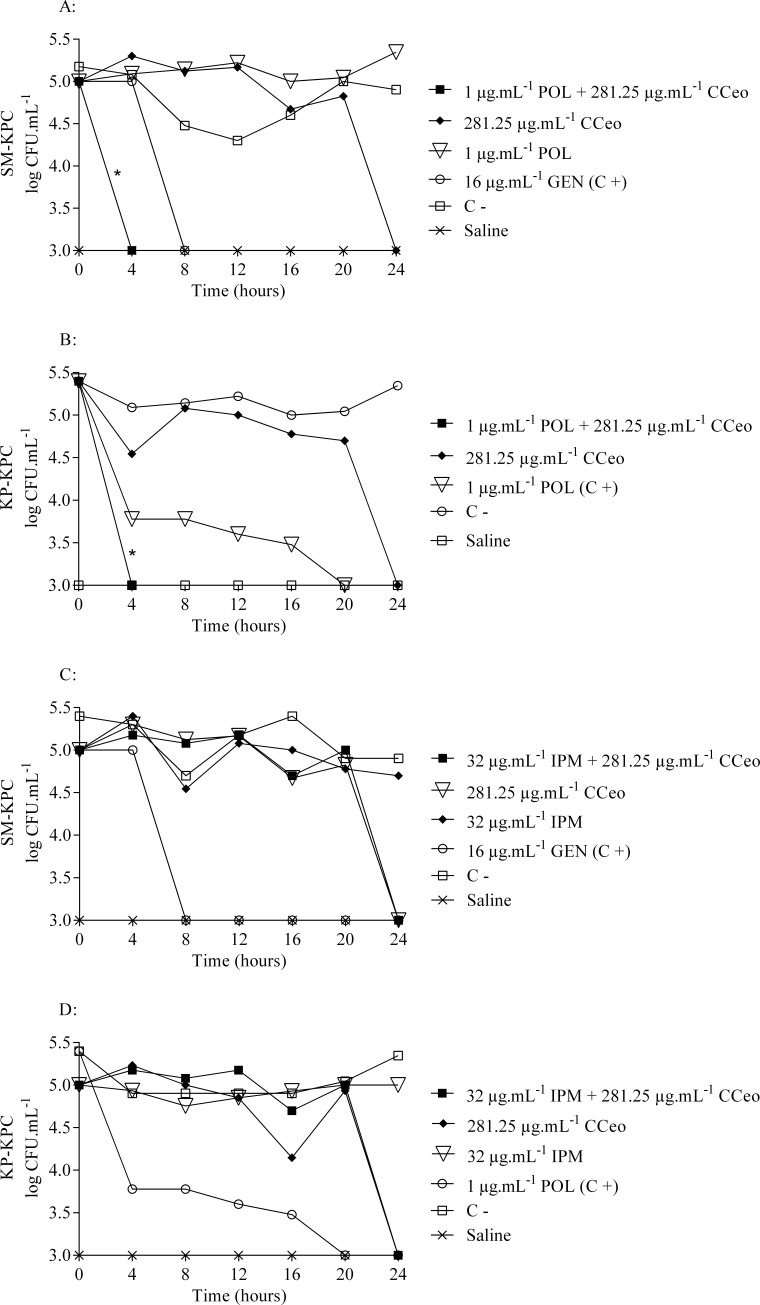
Time-kill curves of the studied carbapenemase-producing strains. (A) CCeo x polymyxin B against SM-KPC. (B) CCeo x POL against KP-KPC. (C) CCeo x IPM against SM-KPC. (D) CCeo x IPM against KP-KPC. C+: positive control; C-: negative control (SM-KPC or KP-KPC and BHI–Brain Heart Infusion broth); CCeo: *C*. *cassia* essential oil; GEN: gentamicin; IPM: imipenem; POL: polymyxin B. Linear regression slopes comparison, *: p < 0.05 comparing to C+.

The survival curves of the strains showed a decrease in viable cell counts over time when CCeo was in association with polymyxin B (Fig 1). CCeo + polymyxin B promoted decreases in cellular count in a range of 5 log_10_ CFU.mL^-1^. Considering the time of cell death, CCeo inhibited KP-KPC and SM-KPC strains within 24 h. Moreover, CCeo + polymyxin B reduced the bacterial load to undetectable levels within only four hours of treatment, which was faster than polymyxin B (20 h) (p < 0.05) and gentamicin (8 h) (p < 0.05) for KP-KPC and SM-KPC, respectively. For KP-KPC, CCeo + polymyxin B time-kill curve presented statistical difference against the negative control (p < 0.05) but not from POL curve (p > 0.05) showing that the synergy CCeo + polymyxin B is as effective as polymyxin B, an used antibiotic against this carbapenemase-producing strain. For SM-KPC, the CCeo + polymyxin B time-kill curve was significantly different from the polymyxin B curve (p < 0.05). The combination of CCeo and imipenem was unable to reduce the bacterial load (p > 0.05). For all treatments, imipenem had no activity within 24 h. The control antibiotics (polymyxin B and gentamicin) successfully inhibited both strains before 24 h. Saline was used as negative control, for which no growth was observed. The effect of the addition of Tween-80 (0.5%) as an oil solubilizer to the antibacterial assays did not interfere with bacterial growth.

**Fig 2:**
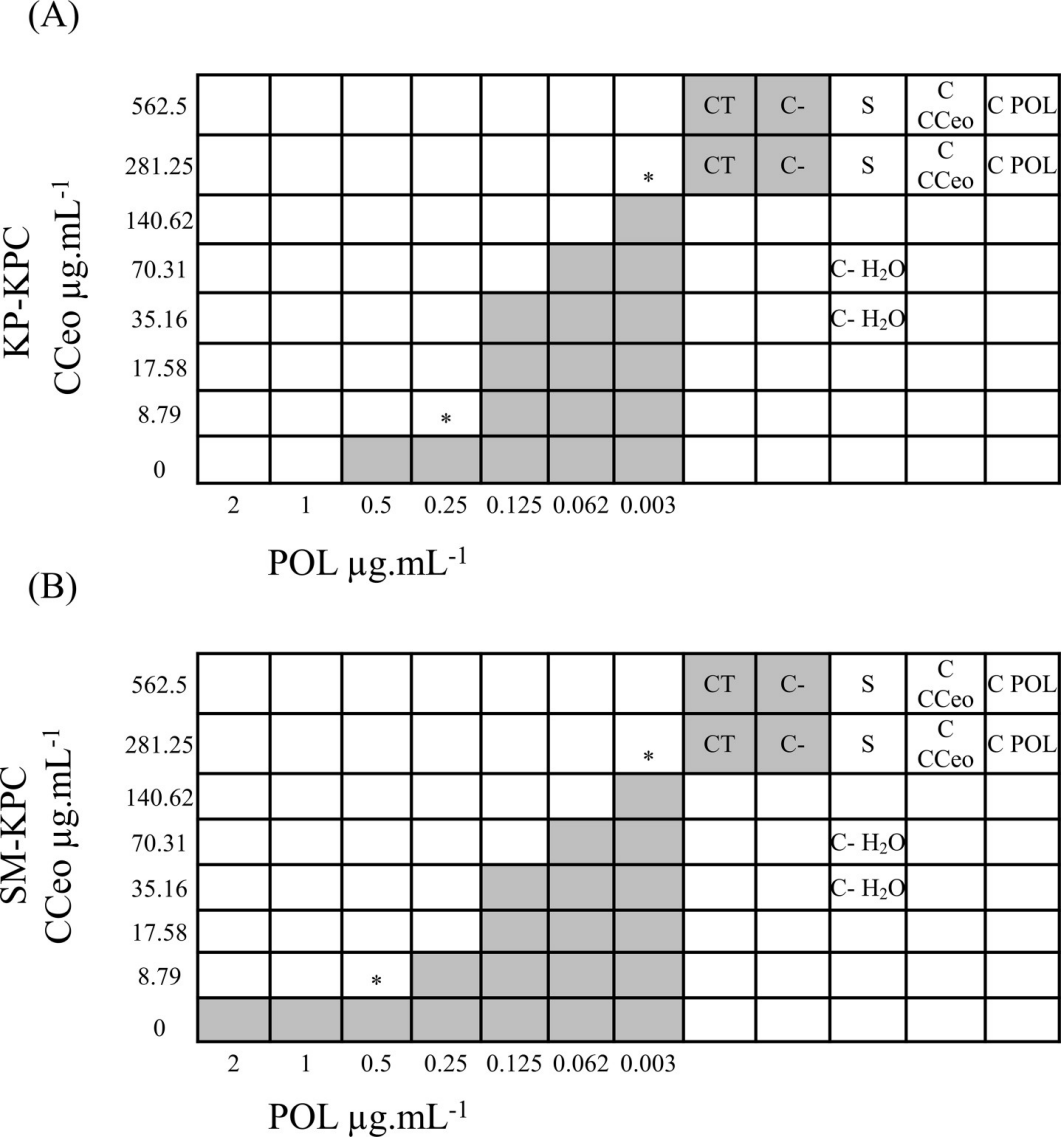
Checkboard assays with CCeo and POL against the studied carbapenemase-producing strains. (A) KP-KPC. (B) SM-KPC. Shading: visible growth; *: optimal association concentrations; CT: tween 80 control; C-: negative control; S: sterility control of saline; C- H_2_O: water control; C CCeo: sterility control of *C*. *cassia* essential oil; C POL: sterility control of polymyxin B.

In the checkboard assay, 49 different combinations of CCeo and polymyxin B were tested for each strain, ranging from twice the MIC to several dilutions below the MIC. The FIC index values were calculated by considering the lowest combinations of CCeo and polymyxin B in which there was no visible growth. The FIC index values were 0.006 for both KP-KPC and SM-KPC. A 320-fold reduction of the polymyxin B MIC was able to inhibit the growth of the strains (Fig 2). Checkboard assay was not performed using imipenem because the time-kill curves did not prove synergism.

**Fig 3:**
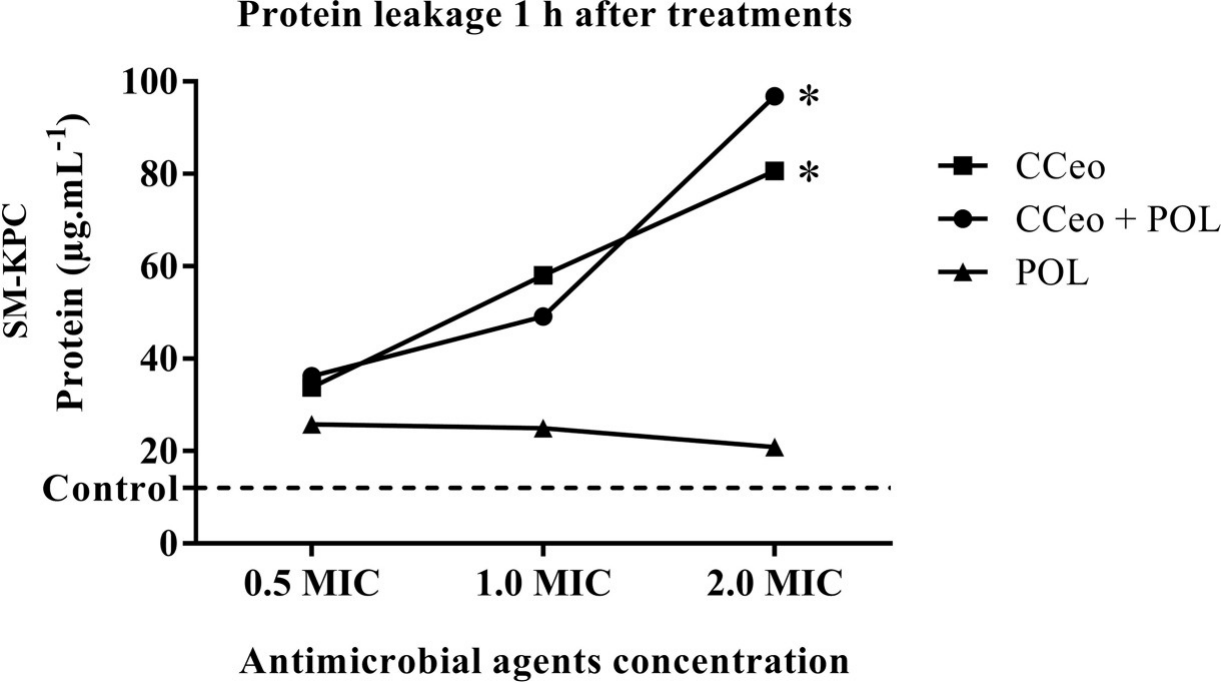
Protein leakage from SM-KPC after 1 hour of CCeo and CCeo + POL treatments. CCeo (MIC, 281.25 μg.mL^-1^), CCeo + POL (MIC CCeo, 281.25 μg.mL^-1^ + MIC POL, 1 μg.mL^-1^) and POL (MIC, 1 μg.mL^-1^). Control: saline with SM-KPC. CCeo: *C*. *cassia* essential oil; MIC: minimal inhibitory concentration; POL: polymyxin B. Linear regression slopes comparison, *: p < 0.05 comparing to POL.

When SM-KPC was treated with increasing concentrations of CCeo and CCeo + polymyxin B, the cells externalized the proteins. The protein leakage from the bacteria in the negative control at 1 h resulted in 12.0 μg.mL^-1^. At a sub-inhibitory concentration (0.5 × MIC), 33.8 μg.mL^-1^ of protein was present in the supernatant of CCeo treatment and 36.2 μg.mL^-1^ for CCeo + polymyxin B, both at 1 h. With CCeo treatment (2 × MIC), 80.6 μg.mL^-1^ of protein was present in the supernatant. With CCeo + polymyxin B (1 h), 96.8 μg.mL^-1^ of protein was present in the supernatant, showing an increase in the protein leakage levels. The concentrations of externalized proteins when cells were treated with CCeo and CCeo + polymyxin B differed significantly from cells treated only with polymyxin B (p < 0.05). Polymyxin B alone did not show any leakage of protein (Fig 3). In addition, no difference was observed in the protein leakage between the time-points studied (1, 2 and 4h). Protein leakage after 1 h in all treatments was the time-point chosen to be represented graphically because the bacterial cells are still viable to grow in the culture media at this time.

**Fig 4:**
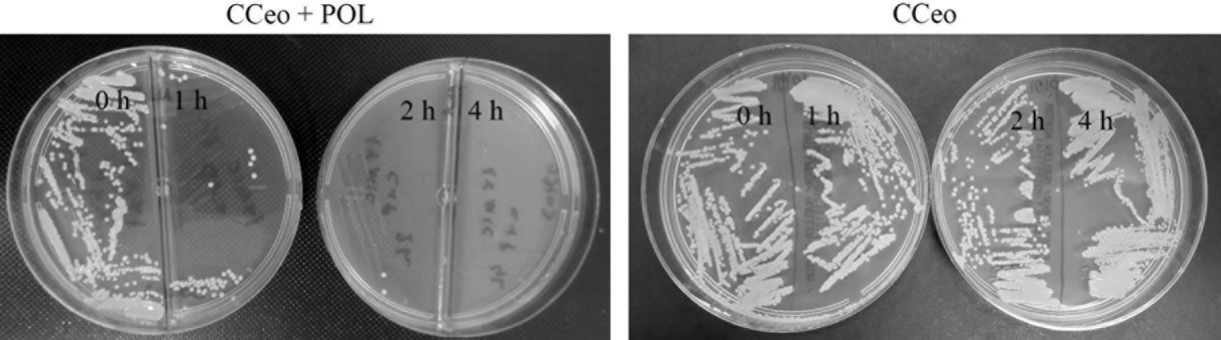
SM-KPC growth after CCeo and CCeo + POL treatments. Müller-Hinton agar plates seeded with 0 h, 1 h, 2 h, and 4 h aliquots of SM-KPC treated with CCeo + POL 1 x MIC and CCeo 1 x MIC.

When the cells were treated with CCeo + polymyxin B, Müller-Hinton agar plates showed abundant growth at 0 h, an expressive growth reduction at 1 h, only 2 CFU at 2 h, and a total inhibition at 4 h. No difference in bacterium growth at 0, 1, 2, and 4 h was observed when the cells were treated with CCeo alone (Fig 4), consistent with the time-kill assay results.

The 16S rRNA transcripts were reduced in SM-KPC cells after treatment CCeo + polymyxin B, as verified by RT-qPCR, confirming a decrease in the bacterial cell number (S2 Fig). The *arnB* transcripts were present before and after treatment with CCeo + polymyxin B. Also, *arnB* gene expression was observed after treatment with polymyxin B (S3 Fig).

## Discussion

As far as we know, there are no studies on the antibacterial action of *C*. *cassia* alone or in combination with antibiotics against carbapenemase-producing Enterobacterales bacteria. Here, *C*. *cassia* was able to inhibit two strains of Gram-negative bacteria resistant to almost all classes of clinical antibiotics, either alone or in combination with polymyxin B. Antibacterial assays demonstrated the potential of action of CCeo against KP-KPC and SM-KPC (MIC of 281.25 μg.mL^-1^). Trans-cinnamaldehyde is the main component with antibacterial action of CCeo, and showed the same MIC as CCeo. Following a new tendency to study the whole plant instead of single compounds [29], assays were conducted with CCeo as a whole. CCeo + polymyxin B was found to be faster than polymyxin B or gentamicin in inhibiting KP-KPC and SM-KPC. This susceptibility was independent of the acquired resistant gene, since KP-KPC expressed *bla*_KPC-2_, and SM-KPC expressed *bla*_KPC-2_ and *bla*_IMP-10_, indicating a mechanism of action not related to the genus, species, or resistance genes. The FIC index value of CCeo and polymyxin B in combination confirmed the synergistic effects between these two agents for KP-KPC and SM-KPC. This indicated that the best dosages of the antimicrobial agents where obtained when these compounds were used in combination, providing a basis for the use of different combinations in the development of alternative treatments.

Imipenem and polymyxin B were chosen for synergism confirmatory tests because they are used clinically for the therapeutic treatment of Gram-negative infections. The doses of CCeo and antibiotics in this study were selected according to the MIC values presented by the strains studied. For carbapenem-resistant bacteria, polymyxin B alone or in combination with other antibiotics is currently the treatment of choice for these infections [30] and this study´s aim was to reach the lowest doses of the antimicrobial agents tested in synergism for an efficient alternative treatment. As the species *Serratia marcescens* has intrinsic resistance to polymyxins, the experimental polymyxin B concentration used as MIC for SM-KPC was 1 μg.mL^-1^, which was the same as the polymyxin B MIC for KP-KPC. The widespread distribution of carbapenemase-producing Enterobacterales has resulted in the increased use of this drug, with the inevitable risk of developing resistance. However, the use of polymyxin is associated with a high incidence of toxicity, such as nephrotoxicity and neurotoxicity [31]. Despite the polymyxins toxicity, when there is no other pharmacological option for treatment, this dosage is administered in patients who present infections with bacteria resistant of carbapenem [32]. The possibility of reducing the necessary dose for treatment with polymyxin B, when associated with CCeo, is a important result since the polymyxin B dose is directly related to its toxicity when used therapeutically. Our study demonstrates that when polymyxin B is associated with CCeo there is a drastic reduction of 320-fold in the dose required for *in vitro* treatment of the studied multidrug-resistant strains, which brings a benefit to the patient who will not have to receive higher doses of a toxic medication. Moreover, lower doses are expected to reduce the selective pressure upon carbapenemase-producing bacteria, thereby minimizing the appearance of resistance [30]. Concerning CCeo toxicity, the major components of *C*. *cassia* are considered to be non-toxic and safe agents with no acute or chronic toxicity, no mutagenicity or genotoxicity, and no carcinogenicity detected in mammalian studies [33].

The synergism results in our study also demonstrate that a dose of 8.79 μg.mL^-1^ of *C*. *cassia* essential oil is able to inhibit the growth of the multi-resistant strains tested. Cinnamaldehyde has been reported as the main component in CCeo and studies showed that Cinnamaldehyde absorption appears to be rapid and complete in mice [34] and in humans. The elimination of cinnamaldehyde by two adult volunteers, who had received a single oral dose of 0.7 mg/kg, was rapid with 100% recovered in the urine within 8 h [35]. Humans also clear systemically available cinnamic acid quickly. Eleven adult human volunteers received single intravenous doses of cinnamic acid, equivalent to 5 mg/kg bodyweight. Plasma was cleared of cinnamic acid (cinnamaldehyde is converted to cinnamic acid in organism) within 20 min [36]. Acute toxicity, oral LD_50_ values for cinnamaldehyde in rats and guinea pigs have been reported as 2220 mg/kg and 1160 mg/kg, respectively [33]. Mutagenicity and genotoxicity in mammalian, there was no evidence of an increase in unscheduled DNA synthesis when rats were administered up to 500 mg/kg bodyweight by intraperitoneal injection in a micronucleus assay [37]. The CCeo oil are intended to be used at low levels of exposure relative to doses that elicit adverse effects in laboratory animals via systemic exposure. A *C*. *cassia* oral safe dose is 0.7 mg/kg/day [33], and the bio-accessibility of *C*. *cassia* by oral route was approximately 79% [38]. The minor *C*. *cassia* essential oil dose, demonstrated in our study (8.79 μg.mL^-1^), able to inhibit the growth of the multi-resistant strains tested when associated with polymyxin B is lower than the literature describes as safe concentrations of *C*. *cassia* for humans.

In this study, CCeo was found to be efficient as an excipient for polymyxin B, reducing the dose of antibiotic required for treatment. Thus, micro-emulsions and nano-emulsions synthesized using CCeo may be used as drug delivery systems. Due to their potent antibacterial activity and physicochemical properties, these drug delivery systems could be used to improve the treatment options for human diseases and provide better delivery vehicles for drugs with low bioavailability, thereby decreasing drug toxicity and prolonging the usable life of current antimicrobial drugs [39]. Nanostructured systems have been designed intending essential oils encapsulation as approach to enhance their bio-availability and bio-efficacy as a result of high cellular uptake and controlled release delivery. According to the literature, polymer-based nano-carriers are extensively used for this purpose and no significant cytotoxicity on the normal growth and the development of cultured diploid human cells were observed [40].

The concentrations of leaked proteins after sub-inhibitory (0.5 × MIC) treatments were 2.8-fold (CCeo) and 3.0-fold (CCeo + polymyxin B) higher than the negative control at 1 h. These sub-inhibitory treatment results indicated that the bacterial cells start to leak proteins when they are exposed to CCeo. However, the cells are still viable to grow in culture media. The protein leakage indicates an affected membrane [9] is still able to allow the entrance of polymyxin B to conclude the lysis of the bacterial cell membrane. At 1 × MIC and 2 × MIC of the CCeo and CCeo + polymyxin B treatments, protein leakage was higher. However, late intracellular content extravasation may occur due to other apoptosis pathways being activated [7]. It is possible to assume that CCeo induced concentration-dependent damage in cell membranes, destabilized the outer membrane of the SM-KPC, allowing polymyxin B to enter the periplasm of the cell, disrupting outer membrane integrity, with leakage of cellular contents, and cell death [7]. Moreover, it has already been shown that cinnamon reduces the negativity of the electric charge of *E*. *coli* cell membranes, allowing antibiotics to access the penicillin-binding proteins to induce of cell death [27].

A low FIC value index indicates the low MICs of CCeo and polymyxin B in the inhibition of SM-KPC growth. The mechanisms of action of this synergistic effect could involve CCeo blocking the SM-KPC strategies for resistance to polymyxin B or increasing the outer membrane permeability, allowing polymyxin B to enter the bacterial periplasmic space. The resistance strategies of *S*. *marcescens* to polymyxins include modifications on their LPS, which normally have negative charges and are the initial targets of polymyxins [4]. Several bacterial cell injuries have already been reported for bacteria treated with cinnamon essential oils [27] and cinnamaldehyde [41]. In this study, the expression of the *arnB* gene was observed in SM-KPC before and after treatment with polymyxin B. It is known that polymyxin B can upregulate the *arnB* expression in *S*. *marcescens*, indicating that the bacterial cell is able to sense the presence of polymyxin B and undergo a positive feedback reaction by enhancing the *arnB* expression in order to promote better protection against an imminent drug threat [4]. These results confirm the hypothesis that CCeo disturbs the bacterial outer membrane allowing polymyxin B to enter the cell since, when it was associated with polymyxin B, CCeo was able to inhibit *arnB* overexpression.

The findings presented here are of great significance given the current importance of polymyxin B in clinical practice and an increase in the generalized bacterial resistance to this drug. However, this research was limited to *in vitro* studies. Since our findings were promising with KP-KPC and SM-KPC, strains classified with “Critical” priority pathogens on the list for the research and development of new antibiotics by The World Health Organization (WHO) [42], the development of research with another clinically relevant bacteria, should be extended to *in vivo* investigations, including models of infection.

## Conclusion

CCeo was able to inhibit KP-KPC and SM-KPC and was able to exert this inhibition in combination with polymyxin B at a reduced antibiotic dose. The results presented here, therefore, provide a basis for further *in vivo* and clinical studies for the development of novel treatments using a combination of CCeo and polymyxin B for use against multidrug resistant strains.

## Supporting information

S1 Fig The chromatographic profile obtained with high resolution gas chromatography for *C*. *cassia* oil. AGILENT 7820A Gas Chromatograph. Column: HP-5 30 m x 0.32 mm x 0.25 μm (AGILENT). Temp.: Column: 70°C (0 min), 3°C / min at 240°C. Injector: 240°C Split: 1/50. FID detector: 250°C. Vol. injection time: 1 ul (conc 1.0% in chloroform). Vany Ferraz, Chemistry Department–UFMG (Universidade Federal de Minas Gerais). Requested by Ferquima (Vargem Grande Paulista, SP, Brazil).
(TIF)

S2 Fig Relative expression of 16S rRNA *S*. *marcescens* gene by RT-qPCR. Green lines: *S*. *marcescens* without CCeo + POL treatment; blue lines: *S*. *marcescens* after 1 hour with CCeo + POL treatment; red lines: negative control. Electroforese gel: RT-qPCR products. RFU: Relative fluorescence units.
(TIF)

S3 Fig RT-PCR detection of *arnB* mRNA in *S*. *marcescens* with CCeo + POL and POL treatments. (A) Agarose gel electrophoresis for *arnB* mRNA (169 bp) stained with GelRed—CCeo + POL treatment. Lane 1: molecular weight marker Ludwig 50 x (50 bp); lane 2: negative control; lane 3: before treatment; lane 4: after 1 hour of treatment. (B) Agarose gel electrophoresis for *arnB* mRNA (169 bp) stained with GelRed–POL treatment. Lane 1: molecular weight marker Ludwig 50 x (50 bp); lane 2: negative control; lane 3: before treatment; lane 4: after 1 hour of treatment.
(TIF)
