# Supplementary figures and images for: Synergistic effects of Cinnamomum cassia L. essential oil in combination with polymyxin B against carbapenemase-producing Klebsiella pneumoniae and Serratia marcescens
Source: PLoS One. 2020 Jul 23;15(7):e0236505. doi: 10.1371/journal.pone.0236505 (PMC7377461; doi:10.1371/journal.pone.0236505)

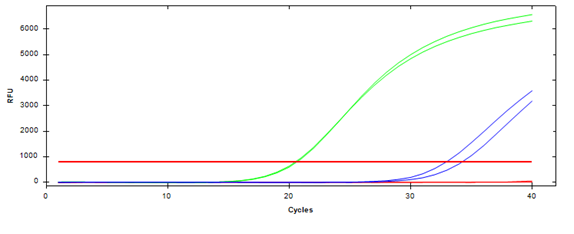

Supplement: S2 Fig — Green lines: S. marcescens without CCeo + POL treatment; blue lines: S. marcescens after 1 hour with CCeo + POL treatment; red lines: negative control. Electroforese gel: RT-qPCR products. RFU: Relative fluorescence units. (TIF) [file pone.0236505.s002.tif]

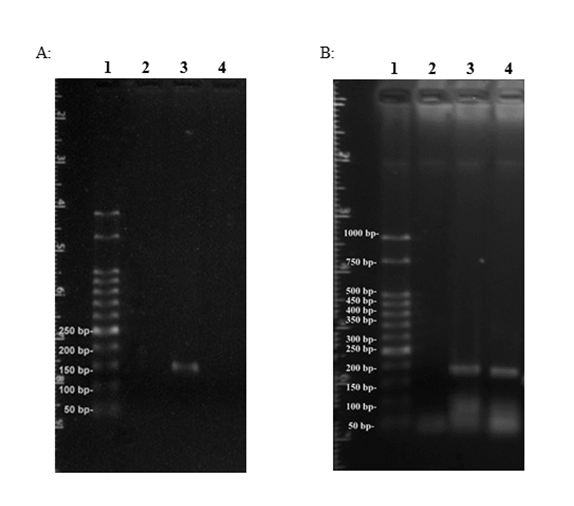

Supplement: S3 Fig — (A) Agarose gel electrophoresis for arnB mRNA (169 bp) stained with GelRed—CCeo + POL treatment. Lane 1: molecular weight marker Ludwig 50 x (50 bp); lane 2: negative control; lane 3: before treatment; lane 4: after 1 hour of treatment. (B) Agarose gel electrophoresis for arnB mRNA (169 bp) stained with GelRed–POL treatment. Lane 1: molecular weight marker Ludwig 50 x (50 bp); lane 2: negative control; lane 3: before treatment; lane 4: after 1 hour of treatment. (TIF) [file pone.0236505.s003.tif]
